# Effect of Delayed Cord Clamping on Respiratory Infection–Related Encounters in the First 6 Months of Life in an Urban Community Hospital

**DOI:** 10.1155/ijpe/1501028

**Published:** 2025-12-12

**Authors:** Ronique Gordon, Neelam Neupane, Leidi Pedraza Gonzalez, Luane Bloomfield, Tashalee McGrath-Blagrove, R. Jonathan Robitsek, Shirley Pinero-Bernardo, Lourdes Cohen, Lily Q. Lew

**Affiliations:** ^1^ Department of Pediatrics, Flushing Hospital Medical Center, Flushing, New York, USA, flushinghospital.org; ^2^ Department of Research Education and Innovation, Medisys Health Network, Jamaica, New York, USA

**Keywords:** delayed cord clamping, hyperbilirubinemia, immunity, infants, infections

## Abstract

**Background/Objective:**

Delayed umbilical cord clamping (DCC) for 30–60 s after birth is recommended for both term and preterm infants. The additional neonatal blood volume rich in stem cells and immunoglobulins may protect the neonate from infections. We aim to compare the effect of DCC in term newborns on hyperbilirubinemia and respiratory infection–related pediatric emergency department (PED) encounters and hospitalizations within the first 6 months of life.

**Methods:**

We conducted a chart review of term infants born between January 1, 2022 and December 31, 2022 and grouped them as either having DCC or not having delayed umbilical cord clamping (nDCC) for 30–60 s after birth. Maternal and newborn characteristics, hyperbilirubinemia, respiratory infection–related PED encounters, hospitalizations, and length of stay in the initial 6 months after birth were compared. Data were analyzed using R software, a *p* value of < 0.05 was considered statistically significant.

**Results:**

Of the 2136 charts reviewed, 659 (31%) were in the DCC group. There were significantly fewer respiratory infection–related PED encounters (*p* < 0.001), fewer hospitalizations (*p* = 0.04), and a 5% lower incidence of hyperbilirubinemia in the DCC group (95% CI: 0.86%–8.6%; *p* = 0.02). The length of stay of each hospitalization was not significantly different between the two groups, *p* = 0.07.

**Conclusions:**

We observed fewer respiratory infection–related PED encounters and hospitalizations in the initial 6 months of life and a lower incidence of hyperbilirubinemia among the infants who had DCC. The increased blood volume and its components appear to be supportive of the neonate′s developing immune system as seen in the lower disease burden up to 6 months of age.

## 1. Introduction

Delayed umbilical cord clamping (DCC) is recommended in vigorous term infants for 30–60 s after birth [1]. Despite well‐documented benefits of DCC in preterm infants, only reduced risk of iron deficiency anemia in the first several months of life has been demonstrated specifically in term infants [2]. The higher blood volume and higher levels of stem cells and immunoglobulins transferred via DCC can provide additional immunity toward infections [3]. Respiratory viral infections in infancy are associated with substantial morbidity and mortality worldwide [4]. While most respiratory infections are viral and self‐limited in nature, respiratory infection–related symptoms in infants account for the majority of pediatric emergency department (PED) visits. Healthcare providers and political decision‐makers need to seek ways to reduce disease burden. Breastfeeding and breastmilk are known to have a protective role against acute respiratory infections. Policies and initiatives have been effective in increasing breastfeeding rates and duration worldwide in the past decade [5, 6]. The addition of DCC can be used as another approach to reduce respiratory infection–related PED visits. However, DCC is not the standard of care globally owing to reports of risk of polycythemia and hyperbilirubinemia [7]. Notwithstanding these concerns, there is a paucity of data on the effect of DCC on immunity in term infants in the literature [8]. To investigate the benefits of DCC, our institution in an urban community studied the impact of DCC on vigorous term infants. The results from our study highlight the potential benefit of fewer respiratory infection–related PED encounters and hospitalizations due to enhanced immunity without increased risk of hyperbilirubinemia in term infants having DCC. Knowledge of the possible association between DCC and improved immunity and lower disease burden provides the rationale to increase the practice of DCC after birth.

## 2. Material and Methods

After the Institutional Review Board approved the study, we performed a retrospective chart review of term infants born between January 1, 2022 and December 31, 2022 at an urban 293‐bed not‐for‐profit community teaching hospital in Queens County, New York City, United States with a dedicated 28‐bed well newborn nursery and a Level III neonatal intensive care unit. All stable full‐term newborns admitted to the well newborn nursery were included in the study.

Term infants, defined as having a gestational age at or greater than 37 weeks, were grouped as either having DCC or not having delayed umbilical cord clamping (nDCC). Neonatal hyperbilirubinemia or hyperbilirubinemia was defined as initiation of phototherapy in accordance with the American Academy of Pediatrics 2004 and 2022 (August) updated guidelines [9]. Data extracted from the electronic medical records included maternal age, body mass index, presence of hypertension and diabetes mellitus, newborn gender, gestational age, birth weight, Apgar scores, incidence of hyperbilirubinemia, number of respiratory infection–related PED encounters and hospitalizations, and length of stay in the initial 6 months after birth. Data were analyzed using R software, Version 4.3.2, with no missing data included in any analyses. Wilcoxon rank‐sum and chi‐square tests compared continuous and nominal variables accordingly; a *p* < 0.05 was considered statistically significant. STROBE guidelines for observation studies were followed.

## 3. Results

**Figure 1:**
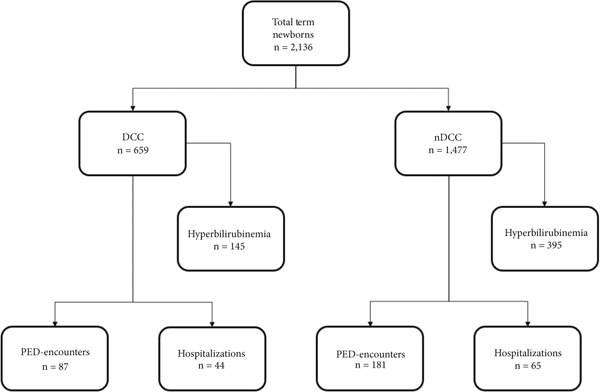
Flow diagram of newborn charts reviewed.

Of a total of 2136 charts reviewed, close to a third of the infants (*n* = 659; 31%) were in the DCC group (Figure 1). There were no significant differences for maternal or neonatal characteristics between the two groups (Table 1). The total number of respiratory infection–related PED encounters and hospitalizations were fewer among those who had DCC compared to those who did not, *p* < 0.001 and *p* = 0.04, respectively. Fewer numbers of encounters in those aged 3–6 months (*p* < 0.001) and fewer hospitalizations in those aged 0–< 3 months (*p* = 0.02) were observed in the DCC group. The length of stay for each hospitalization in days was not different between the groups. The incidence of hyperbilirubinemia requiring phototherapy was 5% lower in the DCC group (22%) compared to the nDCC group (95% CI: 0.86–8.6%; *p* = 0.02) (Table 2).

**Table 1:** Comparing characteristics of the DCC and nDCC groups.

**Characteristics**	**DCC**	**nDCC**	**p** **value**
Maternal: *n* (%)	655 (30.9)	1473 (69.1)	< 0.001 ^∗^
Age (years), mean (SD)	31.1 (5.6)	30.9 (5.7)	0.41
Body mass index (kg/m^2^), mean (SD)	30.1 (5.2)	30.1 (5.0)	0.49
Presence of hypertension (%)	80 (12.2)	196 (13.3)	0.53
Presence of diabetes mellitus (%)	152 (23.2)	378 (25.7)	0.23
Neonatal: *n* (%)	659 (30.8)	1477 (69.2)	< 0.001 ^∗^
Gender: female (%)	335 (50.8)	714 (48.3)	0.30
Gestational age (weeks) mean (SD)	38.6 (0.96)	38.6 (0.97)	0.97
Birth weight (grams) mean (SD)	3231 (413.7)	3235 (446.5)	0.90
Apgar score (1 min) mean (SD)	8.8 (0.55)	8.8 (0.61)	0.53
Apgar score (5 min) mean (SD)	9.0 (0.27)	9.0 (0.29)	0.37

Abbreviations: DCC, delayed cord clamping; nDCC, no delayed cord clamping; SD, standard deviation.

^∗^ *p* < 0.05 was considered statistically significant.

**Table 2:** Comparing variables of the DCC and nDCC groups.

**Variables**	**DCC** **(** **n** = 659**)**	**nDCC** **(** **n** = 1477**)**	**p** **value**
Neonatal hyperbilirubinemia, *n* (%)	145 (22.0)	395 (26.7)	0.02 ^∗^
Total PED encounters 0–6 months, *n* (%)	87 (32.5)	181 (67.5)	< 0.001 ^∗^
Aged 0–< 3 months, *n* (%)	42 (40.8)	61 (59.2)	0.06
Aged 3–6 months, *n* (%)	45 (27.3)	120 (72.7)	< 0.001 ^∗^
Hospitalizations 0–6 months, *n* (%)	44 (40.4)	65 (59.0)	0.04 ^∗^
Aged 0–<3 months, *n* (%)	34 (37.8)	56 (62.3)	0.02 ^∗^
Aged 3–6 months, *n* (%)	10 (52.6)	9 (47.4)	0.8
Length of stay (days) mean (SD)	2.4 (1.1)	2.4 (1.7)	0.07

Abbreviations: DCC, delayed cord clamping; nDCC, no delayed cord clamping; SD, standard deviation.

^∗^ *p* < 0.05 was considered statistically significant.

## 4. Discussion

DCC is a process that allows for the passive placental transfusion of warm, oxygenated blood into the newborn [10]. Following the endorsement by the American College of Obstetricians and Gynecologists and the American Academy of Pediatrics of DCC for 30–60 s after birth owing to the benefits to all healthy term newborns, its implementation as the standard of care has been variable worldwide due to a lack of awareness, knowledge, and healthcare access [10]. Evidence of benefits rendered to preterm infants outnumbered those to term infants [11]. The additional neonatal blood volume increases iron stores that may improve physical and neurodevelopmental outcomes, and the increases in stem cells and immunoglobulins may protect the infant from infections [12]. However, the potential risk of polycythemia and hyperbilirubinemia contributed to some of the hesitancy in adapting the recommended intervention worldwide despite ample data affirming the contrary [13]. Nakayama et al. reported only half of the facilities in the United States practiced DCC for healthy newborns [14]. Similar low prevalence rates were seen in studies by Mohammad et al. in Jordan, Chowdhury et al. in India, and Marya and Chetcuti Ganado in the United Kingdom [7, 15, 16]. There is a paucity of data on improved immunity due to DCC in term infants to promote the practice. We linked fewer respiratory infection–related PED encounters and hospitalizations in the first 6 months of life in those having had DCC to better immunity without increased risk of neonatal hyperbilirubinemia. Besides breastmilk and available vaccines, DCC can be a relatively inexpensive strategy to lower disease burden and to support associated short‐term and long‐term outcomes.

After birth, the neonate can receive increased blood volume, stem cells, and immunoglobulins in the process of DCC. There are data on infectious parameters and immunoglobulins after DCC in preterm infants and not in term infants [12]. When the vulnerable newborns are exposed to the multitude of microorganisms during its transition from the intrauterine to extrauterine environment, they are at great risk of viral and bacterial infections due to their immature immune system and lack of immunological memory [17]. Respiratory viral infections are the most common health concerns in infants especially in those less than 1 year of age and are associated with major morbidity and mortality [18]. Vaccines against respiratory infections recommended for mothers or for infants less than 6 months were either not routinely administered or not available at the time of our study [19]. When we stratified the number of encounters and hospitalizations to 0–< 3 and 3–6 months, infants having had DCC did not demonstrate any waning of immunity that occurs typically at 4–6 months of life [20]. Details of each PED encounter and hospitalization including symptoms, virus detection, and seasonality were not reviewed for this study. The data in this study suggest additional benefit for DCC on improving infant immunity against respiratory viral infections. The number of PED encounters and hospitalizations contribute to disease burden and can stress healthcare systems during the peak seasons of respiratory viral infections.

Breastmilk offers immunologic benefits and plays a protective role against respiratory infections [21]. As a designated Baby‐Friendly USA Hospital of the US Baby‐Friendly Hospital Initiative, all stable full‐term newborns regardless of the mode of delivery are immediately placed on the mother′s abdomen or chest during DCC and monitored by the Apgar timer in the delivery suites to facilitate skin‐to‐skin contact, a practice to promote breastfeeding [22]. During our study period, our institution had an exclusive breastfeeding rate of 10% with 88% of infants receiving a combination of breastmilk and formula. Diet history was not obtained in our study but is unlikely to differ significantly between our two groups to affect our results.

Maternal comorbidities such as obesity, hypertension, and diabetes mellitus have been shown to affect the immunity of the offspring negatively [23]. The maternal body mass index, presence of hypertension, and diabetes mellitus were not different between the two groups. The length of stay was also not affected by DCC.

We did not observe an increased risk of hyperbilirubinemia at birth hospitalization in the DCC group, contrary to higher rates reported by some authors [24]. Yang et al. in 2019 reported higher mean peak transcutaneous bilirubin levels and no increase in the need for phototherapy in their small cohort of term neonates having DCC [25]. Our hospital adapted the 2022 American Academy of Pediatrics updated guidelines on managing hyperbilirubinemia using transcutaneous bilirubin levels and higher phototherapy thresholds shortly after its release. Thus, the results on hyperbilirubinemia in the latter part of our study period were in accordance with the revised recommendations. This change in protocol may possibly have affected our results. Nevertheless, a reduction in the rate of hospitalization for jaundice in the first full year immediately after the implementation of the updated guidelines was seen in a multicenter study by Jameel et al. [26]. Reassuring studies refuting the higher potential risk of hyperbilirubinemia and its consequences can enhance the acceptance of DCC as the standard of care.

Several limitations were acknowledged in our study besides its retrospective nature and small sample size in a single center. We designated each PED encounter and hospitalization as a unique event. The number of PED encounters and hospitalizations to neighboring facilities was not included. Thus, our results may underestimate the effects of DCC on respiratory infections. The etiology and seasonality of respiratory infections or maternal vaccine status were not determined in our sample. In addition, data on the use of any intrapartum medications were not collected. All unstable newborns are observed in the neonatal intensive care unit and were excluded from the study. Furthermore, our management of neonatal hyperbilirubinemia changed during our study period in adherence to the updated guidelines. As an observational study, there are inherent confounding biases with no intention to demonstrate causality. The strengths of the study include clinical evidence of a potentially beneficial outcome from DCC without additional risk. Further studies are needed on long‐term ramifications seen with less disease burden and improved immunity.

## 5. Conclusions

We observed an association of DCC with fewer respiratory infection–related PED encounters and hospitalizations in the first 6 months of life and no association of increased risk of hyperbilirubinemia. Our study suggests a link between DCC and strengthened immunity. It is essential to have strategies in place for the management of neonatal hyperbilirubinemia when introducing DCC. Healthcare providers dedicated to the care of newborns and infants should continue to support and encourage DCC when appropriate for its beneficial effects.

## Disclosure

Portions of the manuscript were presented at the Eastern Society for Pediatric Research Annual Meeting 2024, 47^th^ Annual Scientific Meeting of the New York Perinatal Society and Pediatric Academic Societies Annual Meeting 2024. All authors approved the final manuscript and agreed to be accountable for all aspects of the work.

## Conflicts of Interest

The authors declare no conflicts of interest.

## Author Contributions

R.G. was responsible for conception/design; literature search; data acquisition, analysis, and interpretation; and manuscript preparation and revision. N.N., L.P.G., L.B., and T.M‐B. contributed to the design; data collection, analysis, and interpretation; and manuscript preparation and revision. R.J.R. was responsible for conception/design, data analysis and interpretation, and manuscript preparation and revision. S.P‐B. and L.C. supervised the conception/design, data collection, analysis, and interpretation; drafting of the initial manuscript; and editing and revision. L.Q.L. was responsible for conception/design, literature search, data interpretation, manuscript preparation, editing, revision, and submission.

## Funding

No funding was received for this manuscript.

## Data Availability

The data used to support the findings of the study were included in this article.
